# Factors that determine directional constraint in ipsilateral hand–foot coordinated movements

**DOI:** 10.1002/phy2.108

**Published:** 2013-10-20

**Authors:** Kento Nakagawa, Tetsuro Muraoka, Kazuyuki Kanosue

**Affiliations:** 1 Graduate School of Sport Sciences, Waseda University2-579-15 Mikajima, Tokorozawa, Saitama, 359-1192, Japan; 2 Japan Society for the Promotion of ScienceTokyo, Japan; 3 College of Economics, Nihon University1-3-2 Misaki-Cho, Chiyoda-Ku, Tokyo, 101-8360, Japan; 4 Consolidated Research Institute for Advanced Science and Medical Care, Waseda University135-1 Horinouchi, Tokorozawa, Saitama, Japan; 5 Faculty of Sport Sciences, Waseda University2-579-15 Mikajima, Tokorozawa, Saitama, 359-1192, Japan

**Keywords:** Hand–foot coordination, interlimb interaction, kinesthesis, motor control

## Abstract

In performing simultaneous rhythmic movements of the ipsilateral hand and foot, there are differences in the level of stability between same directional (stable) and opposite directional (unstable) movements. This is the directional constraint. In this study, we investigated three factors (“interaction in efferent process,” “interaction of afferent signals,” and “error correction”) proposed to underlie for the directional constraint. We compared the performance of three tasks: (1) coordination of actively moved ipsilateral hand and foot, (2) active hand movement in coordination with passively moved foot, (3) active hand movement not coordinated with a passively moved foot. In each task, both same and opposite directional movements were executed. There was no difference between performance estimated with success rate for the first and second task. The directional constraint appeared in both tasks. Thus, the interaction in efferent processes, which was shown to be responsible for the directional constraint in bimanual coordination, was not involved with the directional constraint of ipsilateral hand–foot coordination. The directional constraint did not appear in the third task, which suggested that “interaction of afferent signals” also had no contribution. These results indicated that “error correction” must be the most critical of these factors for mediating the directional constraint in ipsilateral hand–foot coordinated movements.

## Introduction

Coordination of multilimb movements is required not only in daily life but also for performing sports or playing musical instruments. Previous studies have shown that coordinated multilimb movements are restricted by various constraints (Swinnen 2002). Two upper limbs have been used extensively in the study of these constraints (Kelso 1984; Mechsner et al. 2001; Swinnen 2002; Swinnen and Wenderoth 2004). For example, when both hands are simultaneously moved in the horizontal plane, symmetrical movements are more stable and accurate as compared with parallel movements (Swinnen 2002). Likewise, when humans execute cyclic movements of ipsilateral upper and lower limbs in the sagittal plane, movements in an opposite direction are relatively unstable and inaccurate as compared with movements in the same direction. If movement frequency is gradually increased, at some point the opposite directional movements shift to the more stable and accurate same direction movements (Baldissera et al. 1982; Kelso and Jeka 1992; Carson et al. 1995; Salesse et al. 2005; Muraoka et al. 2013). Although the above mentioned directional constraints of multilimb movements are well recognized, the underlying mechanisms, especially those involved with ipsilateral limb coordination, are not well understood.

In an investigation of the coordination of bimanual movement, the elegant experiments of Ridderikhoff et al. (2005) suggested that “integrated timing,” the temporal coupling of multiple internal timekeepers or oscillators for individual limb movements without afferent feedback, plays a major role in establishing the directional constraint. However, the mechanisms involved with the directional constraint in ipsilateral limb coordination might differ, as there are critical differences between bimanual and ipsilateral limb coordination. For example, homologous muscles are utilized in bimanual coordination but not in ipsilateral limb coordination. Additionally, while the control of bimanual coordination in the horizontal plane is accomplished with an intrinsic reference frame (Swinnen 2002), the control of ipsilateral limbs in the sagittal plane is accomplished by utilizing an extrinsic reference frame (Muraoka et al. 2013). It thus seems likely, that different neural mechanisms underlie bimanual coordination and ipsilateral limb coordination.

This study is focused on what produces the directional constraint in ipsilateral limb coordination, utilizing a similar approach to that of Ridderikhoff et al. (2005). In addition to “integrated timing” (we will refer to this factor as “interaction in efferent signals” in this article) Ridderikhoff et al. (2005) postulated two other factors that might be implicated in the production of directional constraints. One factor involves “phase entrainment” by afferent input from the two limbs (Swinnen et al. 1995; Serrien et al. 2001) (we will refer to this factor as “interaction of afferent signals”), and a second factor is concerned with an intentional process of “error correction” by the central nervous system (CNS) which adjusts the relative phase of two-limb movements utilizing a negative feedback with afferent signals from the two limbs. However, in the case of bimanual movements, these latter two factors were determined to be uninvolved in production of the directional constraint (Ridderikhoff et al. 2005).

**Figure 1:**
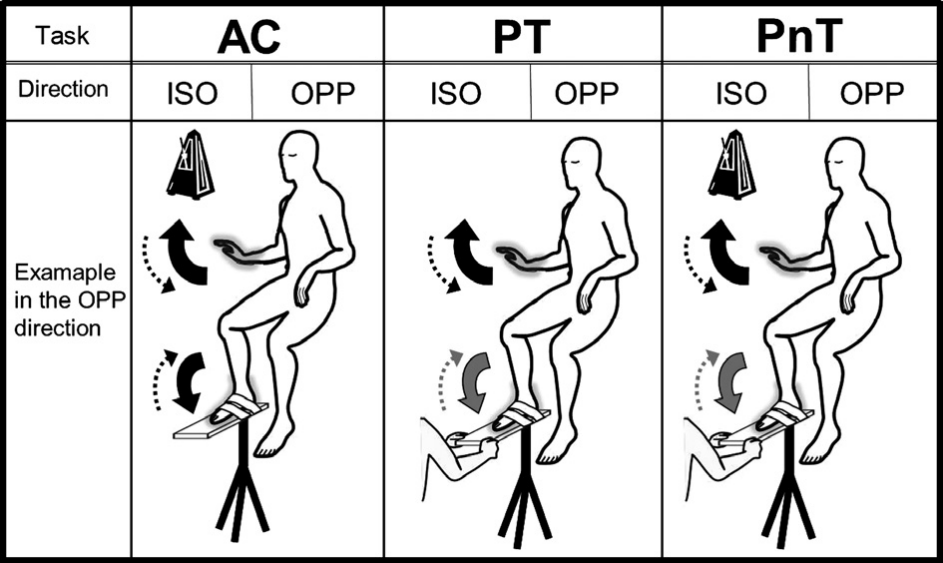
Experimental setup for the three tasks that were performed (AC, PT, and PnT). Each condition had two directions (ISO, the same direction; OPP, the opposite direction). Black arrows indicate active movements executed by the subject, whereas gray arrows indicate passive movements executed by the experimenter. The metronome's presence indicates that the subjects could hear the metronome and thusly guide their pace. AC, active coordination; PT, passive tracking; PnT, passive nontracking.

In this study, we utilized three types of hand–foot cyclic movements (Fig. 1), according to the experimental protocol of Ridderikhoff et al. (2005), and examined whether or not the directional constraint appeared in each movement. The first movement involved “active coordination (AC).” In this situation, both hand and foot were actively moved by the subject. It has been repeatedly reported that directional constraint appears in this kind of task. The second movement utilized “passive tracking (PT).” In this condition subjects only moved one limb, but moved it in coordination with the other limb which was passively moved by the experimenter. The third movement involved “passive nontracking (PnT).” In this mode the subjects actively moved one limb as prescribed to metronome sound. The other limb was passively moved by the experimenter at the same pace, and subjects did not have to attend to hand–foot coordination. These three kinds of movements contain different combinations of the potential factors underlying the directional constraint (Table 1). Thus, by examining in which of the three movements the directional constraint appeared, we would be able to identify the factor responsible for the directional constraint in ipsilateral limb coordination. Our working hypothesis was that the critical factor involved in the directional constraint in the ipsilateral hand–foot coordination differed from the critical factors of bimanual coordination (Ridderikhoff et al. 2005).

**Table 1:** Mapping of the tasks (AC, PT, and PnT) and the three proposed factors in hand–foot coordination

		Possible factors		
		
Task		(1) Interaction in efferent process	(2) Error correction	(3) Interaction of afferent signals
AC	Actively coordinating hand and foot movements	**○**	**○**	**○**
PT	Actively tracking movement of hand to passive moved foot	X	**○**	**○**
PnT	Actively nontracking hand movement to passive moved foot	X	X	**○**

Open circles represent the factors that are possibly involved in each task. Crosses indicate the factors that are not involved in each task. AC, active coordination; PT, passive tracking; PnT, passive nontracking.

## Methods

### Subjects

Nine healthy right-handed adults (27 ± 2 years old) participated in the experiments. Written, informed consent was obtained from all subjects. This study was approved by the Human Research Ethics Committee of the Faculty of Sport Sciences, Waseda University. The experiments were conducted in accordance with the Declaration of Helsinki.

### Experimental setup

In this experiment movements of the right hand and right foot were analyzed. Subjects sat comfortably in a chair with the forearm on an armrest and fixed in a prone position. Additionally, the foot was taped to a wooden board attached to a tripod which allowed the subject's foot to be moved in the sagittal plane (Fig. 1). Angular displacements of the wrist and ankle were measured at 1 kHz using electrical goniometers (SG150; Biometrics, U.K.). The joint signals were low-pass filtered with a cutoff frequency of 10 Hz. All signals were converted into a digital format with an analog-digital converter system (Power lab; AD Instruments, Japan) and stored on a computer.

### Tasks

For all tasks, the subjects kept their eyes closed. The presence of white noise masked the sounds of machines or motions involved with the experiments, but allowed the subjects to hear the metronome. Three tasks were administrated to the subjects; two of them were coordination tasks (AC and PT), and third was a noncoordination task (PnT) (Table 1, Fig. 1). In the AC task, the subject performed active coordinated movements of the hand and foot at a pace indicated by the metronome (2 Hz). In the PT task, passive movements of the foot were produced by an experimenter who heard the metronome through headphones, and the subjects actively moved the hand in coordination with the passively moved foot. The noncoordination task (PnT) involved active movement of the hand by the subject according to the metronome sound and passive movement of the foot by the experimenter. Subjects were instructed to ignore the passive movement of their foot. To the extent possible, the amplitude of the passive movement produced by the experimenter mimicked the amplitude of the AC. Before the experiment, subjects practiced moving the hand and/or foot at the metronome pace (4 Hz) that was actually used in the experiments. Subjects were instructed to make peak extension and peak flexion at each metronome sound, which resulted in a movement frequency of 2 Hz. Subjects were requested to perform 20 cyclic flexion–extension movements of the right hand and the right foot. They were instructed to operate in a range in which they could perform the movement comfortably and smoothly. In the AC task, subjects arbitrarily started the hand and foot movements in the same (AC_ISO) or opposite direction (AC_OPP). In the PT task, the subject was asked to relax the foot muscles. In this condition the subject could not hear the metronome. The experimenter first started to move the subject's foot to the metronome sound from his earphones. Then, the subject started their hand movement after perceiving the passive movement of the foot so that the movements of hand and foot moved in unison (PT_ISO) or in opposite directions (PT_OPP). In the PnT task, subjects were just asked to move their hand to the metronome sound. The experimenter then moved the subject's foot to the same metronome sound in order to synchronize it with the subject's hand movement in the same direction (PnT_ISO), or in the opposite direction (PnT_OPP).

Subjects performed six sessions of six different conditions (three tasks × two directions). They were told that the first session was a practice session and would be eliminated from the analysis. In each session, the order of the six conditions was randomized. Subjects were allowed to rest ad libitum between sessions.

### Data analysis

To evaluate the performance of hand and foot coordination, the relative phase φ between the movements of the hand and foot was calculated for each cycle as φ_*hf*_ = 360°(*t*_*f,i*_ *− t*_*h,*i_)/(*t*_*f,i*+1_ *− t*_*f,i*_), where *t*_*h,i*_ and *t*_*f,i*_ indicate the time of the *i* th peak extension of the hand and foot, respectively (Carson et al. 1995; Ridderikhoff et al. 2005; Volman et al. 2006). In the noncoordination (PnT) task, the relative phase between the movements of the foot and the metronome signals was also calculated as φ_*hs*_ = 2*π*(*t*_*s,i*_ *− t*_*h,i*_)/(*t*_*s,i*+1_ *− t*_*s,i*_*)*, where *t*_*s,i*_ indicates the time of the *i* th signal of the metronome sound. We calculated the standard deviation of the relative phase between the passively moved foot for the PT and PnT. There was no significant difference in this value between ISO and OPP conditions for both tasks. This indicates that the directional constraint was not related to the experimenter who created the passive movement. We defined a relative phase between hand and foot of 0 ± 60° as “in-phase,” that of 180 ± 60° as “antiphase,” and others as “intermediate phase.” When relative phases departed from their target value by “phase wandering,” the posttransition value was rescaled (e.g., 360° = 0°). The proportions of movement cycles classified into these three types of phase were calculated for each trial. A similar evaluation was utilized in previous studies (Carson et al. 1995; Mechsner et al. 2001; Salesse et al. 2005). In addition, the rate of antiphase cycles during OPP conditions and the rate of in-phases during ISO conditions were defined as the “success rate” of each task. The rate of in-phase cycles during OPP conditions was defined as the “entrainment rate.” Moreover, we calculated the difference between the peak extension and flexion angles in each cycle, and this was defined as movement amplitude for the hand and foot. The mean movement frequencies of each trial for hand and foot movements were also calculated.

### Statistical analysis

For all indexes, the equality of variances was first tested by Levene's test. When Levene's test detected significance, a nonparametric analysis was done. That is, Wilcoxon tests for post hoc after Friedman tests were given among tasks. When Levene's test detected no significance, an analysis of variance (ANOVA) was applied. The data of movement frequency, amplitude, and success rate were analyzed by means of a two-way ANOVA with repeated measures (3 tasks [AC, PT, PnT] × 2 movement directions [ISO, OPP]). The proportions of the three modes were analyzed by one-way ANOVA with repeated measures at each mode in each direction. When a significant main effect or interaction was detected, a post hoc Bonferroni test was performed to make a comparison between the tasks. We checked whether the standard deviation of relative phase between movement of the passively moved foot and the metronome sound in the PT and PnT tasks depended on movement direction. This was done by means of a paired *t*-test for each task. All data were expressed as the mean ± SD. Threshold for statistical significance was set to 0.05.

## Results

### Movement frequency and amplitude

The Levene's test showed inequality of variance in the movement frequency of the foot among tasks (*P* < 0.001). Therefore, we conducted Friedman test and it showed significance (*P* < 0.001). However, post hoc analysis did not find any significant difference among tasks. For movement amplitude, Levene's tests showed equality of variance for both hand and foot movements. A one-way ANOVA revealed a significant main effect in the amplitude of the hand (*F*(1,8) = 4.94, *P* < 0.01), but no significant main effect in the amplitude of the foot. The post hoc test indicated that the hand amplitude in AC_OPP (97 ± 38°, range 55–135°) was larger than that of AC_ISO (89 ± 37°, range 55–132°) (*P* < 0.05).

### Qualitative observation in coordination performance

**Figure 2:**
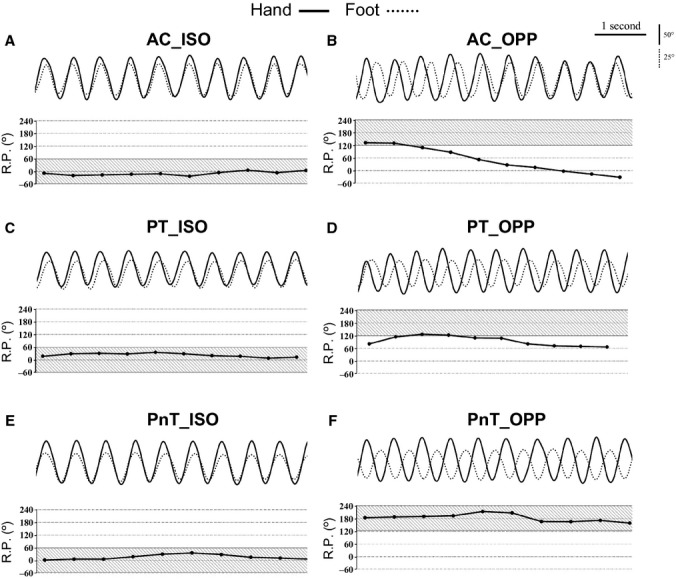
Typical changes in wrist and ankle joint angles, and the relative phases between them in a trial involving AC(A and B), PT(C and D), and PnT tasks (E and F). Left figures indicate the same direction and right figures indicate opposite direction. The black solid and dashed lines show wrist and ankle angle, respectively. The lower graph in each figure shows the relative phase of wrist and ankle angles. The gray area indicates the success range in each condition (ISO, 0 ± 60°; OPP, 180 ± 60°). A–F are AC_ISO, AC_OPP, PT_ISO, PT_OPP, PnT_ISO, and PnT_OPP, respectively. AC, active coordination; PT, passive tracking; PnT, passive nontracking; ISO, the same direction; OPP, the opposite direction.

Figure 2 shows typical angular displacements of hand and foot for part of a trial in each condition for one subject. In the same direction condition (ISO), all three tasks could be performed stably and accurately (Fig. 2A, C, and E); that is, relative phase was maintained around 0° throughout the trial. In contrast, in the opposite direction condition (OPP) movements of the hand and foot were not necessarily stable and stability depended on the particular task. In the AC_OPP condition, hand and foot movements started with a relative phase of 120°, but the phase drifted toward 0° (same directional movements) at the end of the trial (Fig. 2B). This kind of phase transition of movements from the antiphase (opposite direction) to in-phase (same direction) occurred frequently in most of the subjects. The performance of the PT_OPP condition (Fig. 2D) was also unstable; the relative phase fluctuated throughout the trial and did not stabilize in either in-phase or antiphase. In contrast, in the PnT_OPP condition (Fig. 2F) hand and foot movements were maintained around 180°; there was almost no phase transition or phase wandering.

**Figure 3:**
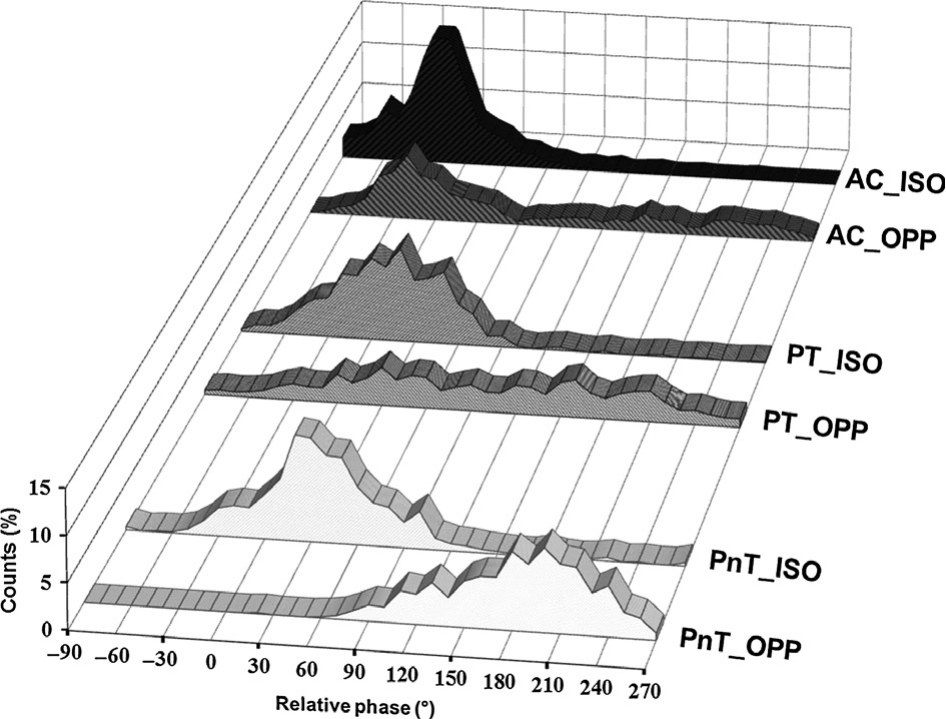
Histogram plots of proportion of the relative phase counts in each tasks. The top two rows, AC; middle two rows, PT; bottom two rows, PnT. AC, active coordination; PT, passive tracking; PnT, passive nontracking.

Similar characteristics were also observed in the distribution of relative phases in all subjects as shown in Figure 3. In all three ISO conditions, the relative phases have a peak around 0° with a small variability. In contrast, the distribution patterns appear quite different among the three OPP conditions. In the AC_OPP condition, although a large variability is apparent, the relative phase has a peak around 0°, indicating that movement transition from opposite direction to same direction occurred frequently. The phase distribution in the PT_OPP condition has no peak, but large variability. These results indicate that the directional constraint appears in the AC and PT tasks. In the case of the PnT_OPP condition, the distribution of relative phase is narrow. Note that the relative phase converged around the target both in the PnT_ISO (0°) condition and in the PnT_OPP (180°) condition.

### Quantitative analysis of performances in the three modes

**Figure 4:**
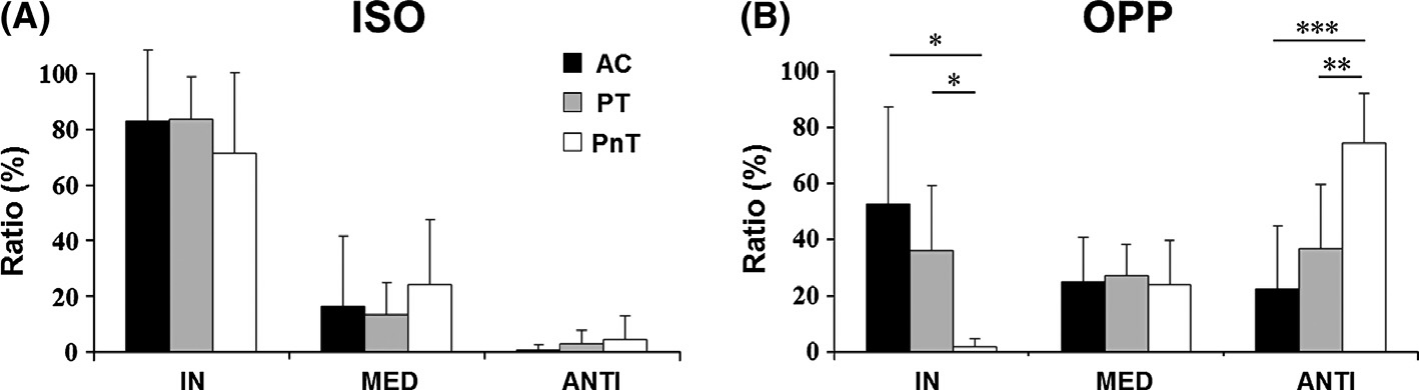
Ratios of antiphases (ANTI: 180 ± 60°), in-phases (IN: 0 ± 60°), and intermediate phases (MED: other phases). (A) same directional tasks (ISO) and (B) opposite directional tasks (OPP). Black, gray, and white bars are AC, PT, and PnT, respectively. Error bars indicate the standard deviation. Significant difference between the tasks are expressed with asterisk marks (*P < 0.05; **P < 0.01; ***P < 0.001). ISO, the same direction; OPP, the opposite direction; AC, active coordination; PT, passive tracking; PnT, passive nontracking.

Ratio of in-phases (IN: 0 ± 60°), antiphases (ANTI: 180 ± 60°), and intermediate phases (MED: other phases) is shown for three tasks (Fig. 4). There was inequality in variance only in the in-phase mode of the OPP task (*P* < 0.001). Subsequent Friedman and Wilcoxon tests detected that the proportion of in-phase (entrainment rate) of AC and PT of the OPP tasks were significantly higher than that of PnT (AC vs. PnT: *P* < 0.05; PT vs. PnT: *P* < 0.05). The other tasks were tested by one-way ANOVA because they were confirmed to have an equality of variance. A significant main effect was found only in the antiphase mode of the OPP task (*F*(2,16) = 22.85, *P* < 0.001). A post hoc test showed that the proportion of antiphase (success rate) of AC and PT were significantly lower than PnT (AC vs. PnT: *P* < 0.001; PT vs. PnT: *P* < 0.01) in the OPP tasks. For the ISO direction, a one-way ANOVA detected no difference among the three tasks in each mode. Additionally, no significant difference between AC and PT was found in any phase category in either the OPP or the ISO tasks (Fig. 4).

The success rate of the OPP task was smaller than that of the ISO task in AC (*P* < 0.01) and PT (*P* < 0.001) condition, which indicates the existence of a directional constraint (Table 2). However, there was no difference in the success rates between the ISO and OPP tasks in the PnT condition.

**Table 2:** Success rate (ISO, in-phases of the ISO direction; OPP, antiphase of the OPP direction) for each task

	ISO	OPP	
AC	83.0 ± 25.6	22.3 ± 22.7	*
PT	84.0 ± 15.6	37.1 ± 23.0	**
PnT	71.5 ± 28.7	74.3 ± 17.8	n.s.

Significant differences between the ISO and OPP are expressed with asterisk marks on the far right (*P < 0.01; **P < 0.001). AC, active coordination; PT, passive tracking; PnT, passive nontracking; ISO, the same direction; OPP, the opposite direction.

## Discussion

Coordinated cyclic movements of the ipsilateral hand and foot are more unstable and inaccurate when they are moved in opposite directions than when they are moved in the same direction. This phenomenon is referred to as the directional constraint (Baldissera et al. 1982; Kelso and Jeka 1992; Carson et al. 1995; Salesse et al. 2005; Muraoka et al. 2013). The objective of the present research was to examine the relative contributions of three factors that are potential mediators of the directional constraint. These factors have been previously proposed (Swinnen et al. 1995; Serrien et al. 2001; Ridderikhoff et al. 2005) and can be concisely described as an “interaction in efferent process,” an “interaction of afferent signals,” and an “error correction.” We systematically analyzed kinematic data of the three tasks (AC, PT, and PnT) to which the factors were presumed to contribute with different combinations (Table 1). The main result of the present experiment was that the directional constraint appeared in the AC and PT, and not in the PnT. This result might not be related to movement frequency or amplitude because there were no differences in these parameters of movement for either the hand or foot among the tasks except that the hand amplitude in AC_OPP was larger than that in AC_ISO by 8°. As this difference was much smaller than the individual variation (55–135°) the amplitude difference is not likely to be the cause of directional constraint.

Among the three possible mechanisms that might underlie the directional constraint, the interaction in efferent process could appear only in the AC because subjects have to control two limbs voluntarily in the AC, and not in the PT or PnT (Table 1). Therefore, if the directional constraint appeared only in the AC, interaction in efferent process would be the main factor that mediated the directional constraint. Furthermore, subjects should correct errors in the relative phase between the movements of two limbs in the AC and PT, but not in the PnT because in the PnT subjects did not actively track the passive movement. Therefore, if the directional constraint appeared in the AC and PT but not in the PnT, the process of “error correction” would be mainly responsible for the directional constraint. Finally, if the directional constraint persisted even in the PnT, it would be caused by an “interaction of afferent signals” from the two limbs because the other two factors did not exist in the PnT.

Interestingly, there was no difference between PT and AC in the success rate or in the entrainment rate. In other words, the directional constraint occurred similarly in both AC and PT. Therefore, the interaction in efferent processes should play no major role in the directional constraint in the ipsilateral hand–foot coordinated movement. In previous studies, the performance of ipsilateral hand–foot coordinated movement was shown to deteriorate when a third limb was passively moved (Swinnen et al. 1995; Serrien et al. 2001). These authors suggested that kinesthetic afferent signals from the passively moved limb must have interfered with the movements of the other two limbs. Similarly, if the afferent signals from the passively moved foot had intervened in the hand movement of the PnT in this study, and if this had produced the directional constraint, the performance of PnT_OPP would have been worse than that of PnT_ISO. However, in both PnT_ISO and PnT_OPP the performances showed similarly high success rates, and almost no entrainment occurred even in the OPP conditions (Fig. 4). This indicates that the afferent signals from the passively moved foot did not interfere with active movement of the ipsilateral hand even if the two appendages moved in opposite directions. These data indicated that there is little or no contribution of interaction of afferent signals to the directional constraint.

The fact that the directional constraint of hand–foot coordination was not observed in the PnT, but was in the PT suggests that “error correction” could be the major factor underlying the directional constraint (Table 1) because the difference between the PT and PnT was whether error correction was accomplished or not. However, it should also be taken into consideration that the metronome guide existed in the PnT and not in PT. Absence of directional constraint in the PnT might be caused by other elements like the “anchoring effect,” which reduces the spatial or temporal variability in rhythmic movement with metronome pacing (Beek 1989; Maslovat et al. 2009). However, although a metronome guide was also utilized in the AC task, the directional constraint appeared in AC, and not in the PnT task. Thus, the supposed “anchoring effect” apparently did not contribute to the directional constraint. Furthermore, it might be that the critical factor for directional constraint is different between AC and PT. For example, the CNS might mainly use sensory feedback (and error correction) in the PT, and switch to a more open loop control system in the AC such that “integrated timing” (Ridderikhoff et al. 2005) was brought into action. But this is not likely to be the case, as it has been demonstrated that afferent information is important for the ipsilateral hand–foot coordinated movement, especially OPP movement (Baldissera et al. 1991; Swinnen et al. 1995).

**Figure 5:**
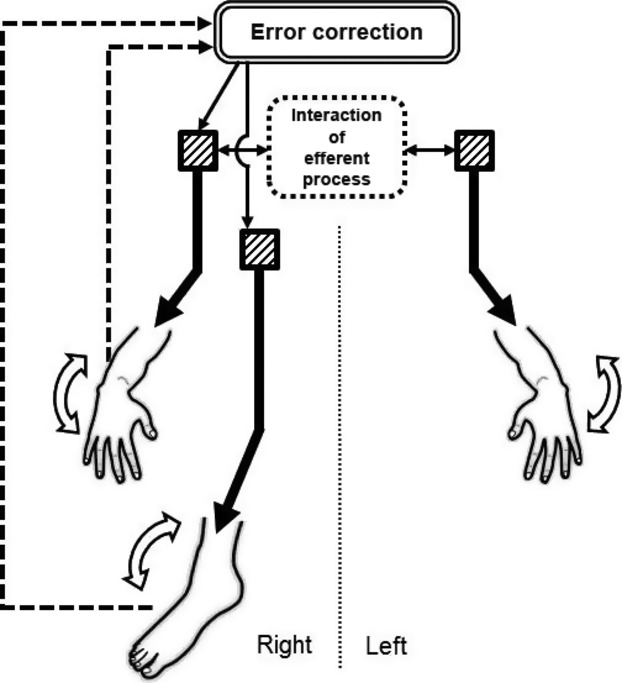
The factors involved in the directional constraint for the ipsilateral hand–foot coordination and for bimanual coordination. The three squares indicate the M1 areas of the right and left hands, and right foot.

The notion that “error correction” is the major factor of directional constraint is inconsistent with Ridderikhoff and colleague's (2005) report that the interaction in efferent process plays an important role for stability in the case of bimanual coordination in the horizontal plane. Their results showed that (1) the directional constraint (the stability difference between symmetric and asymmetric mode in bimanual coordination) was marginal during the coordination of active movement of one hand and passive movement of the contralateral hand (correspondent to our PT task), but prominent during active bimanual coordination (correspondent to our AC task), and (2) the interference strength was similar between symmetric and asymmetric modes in the task that the passive movement was ignored (correspondent to our PnT task). These inconsistencies between the previous and present studies might be attributed to a difference in the combination of two limbs. While there is no anatomical connection between the areas of the ipsilateral hand and foot in the primary motor cortex (M1), a connection exists between the hand areas on both sides via the corpus callosum (Brown et al. 1991). Therefore, efferent signals from the M1 hand and foot areas would separately reach the hand and foot. These signals would not be intermingled with each other. It is plausible that in the case of bimanual movement, when efferent signals are sent from one side of M1 to the effector, signals are simultaneously sent to the contralateral side so as to facilitate the same movement on the other side. Indeed, a cyclic active wrist movement modulates the excitability of the ipsilateral motor cortex corresponding to the phase of the wrist movement. This would result in a higher stability of the same movement for bimanual movements (Carson et al. 2004). Thus, although the directional constraint is similarly observed in bimanual movements and ipsilateral hand and foot movements, the underlying mechanisms are probably different (Fig. 5). Although we considered only cortical mechanism, the present data cannot exclude the involvement of subcortical or spinal mechanisms in the directional constraint or in the differences between bimanual and ipsilateral hand–foot coordination.

In the PT task, the subject's eyes were closed and it was required that subjects move their hand to track the passively moved foot by attending to kinesthetic afferent information from the foot. A directional constraint, very similar to that of two limbs coordination, occurs in the one-limb periodical tracking movement (Buekers et al. 2002; Ceux et al. 2003; Temprado and Laurent 2004). In that situation, arm movement in a direction opposite to periodically moved visual stimulation (such as a point of light) is more difficult than the movement in the same direction. This task is thus equivalent to the PT task of this study in that both require the process of “error correction” between voluntary movement and target movement (visual stimulation or a passively moved foot). Therefore, utilizing “error correction” should induce the directional constraint not only in a two-limb coordinated movement, but also in a one-limb movement, as long as the tasks require the movement of a limb to track a sensory signal (Fig. 5).

The lack of a difference in the success rate among the AC_ISO, PT_ISO, and PnT_ISO tasks could be interpreted to mean that error correction is minimally important in the PT_ISO and AC_ISO, not to mention the PnT which requires no error correction. It has been postulated that opposite directional movement of the ipsilateral hand and foot is more dependent on feedback control than the same directional movement (Baldissera et al. 1991; Swinnen et al. 1995). This view is supported by studies utilizing the reaction time of phonation to an auditory cue during hand–foot coordination, which suggest that fewer attentional loads are needed in same directional movement as compared to opposite directional movement (Hiraga et al. 2004, 2005). Thus, when humans utilize error correction (feedback control) to accomplish the opposite directional movement, it fluctuates and transitions to the more stable same directional movement that requires little error correction. Indeed, functional imaging analysis showed that such areas of cognitive function as the presupplementary motor area, premotor area, and cingulate motor area were additionally activated in opposite directional movement as compared with the same directional movement (Heuninckx et al. 2005; Rocca et al. 2007).

Finally, although this study examined the relative contribution of three factors which have been proposed in previous works, a detailed explanation of the neurophysiological underpinning of the factors was not provided. For this, further neuroimaging and/or electrophysiological studies will be needed.

## Conclusion

This study examined the factors that are responsible for directional constraint of the ipsilateral hand–foot coordinated movements. As the directional constraint appeared similarly in the AC and PT tasks, an “interaction in efferent process” seemed to have no role in the directional constraint. However, the directional constraint was not observed in the PnT task: Subjects could do opposite directional movements as well as same directional movements. This indicates that an “interaction of afferent signals” was also not involved in the directional constraint either. Thus, an “error correction” should be regarded as the critical factor which determines directional constraint in ipsilateral hand–foot coordinated movements.
